# Targeting muscle–vasculature crosstalk in aging through the integrative roles of L-citrulline, leucine, and exercise: focus on muscle metabolism, vascular function, and sarcopenia prevention

**DOI:** 10.3389/fnut.2025.1739173

**Published:** 2026-04-02

**Authors:** Xinyi Lin, Yarui Zhang, Jiangjiang Pu, Yubing Peng

**Affiliations:** 1 Faculty of Health Science and Sport, Macao Polytechnic University, Macao, China; 2 Institute of Brain and Psychological Sciences, Sichuan Normal University, Chengdu, Sichuan, China; 3 Sichuan Vocational College of Culture and Communication, Chengdu, Sichuan, China

**Keywords:** aging, exercise, L-citrulline, leucine, muscle–vasculature, cognitive frailty, successful aging, neurovascular coupling

## Abstract

Aging is characterized by a gradual deterioration in skeletal muscle mass, strength, and vascular functionality, which ultimately leads to the development of sarcopenia and the subsequent loss of physical autonomy. Nutritional and exercise-based interventions that specifically address this interplay may offer viable, non-pharmacological approaches to maintaining both muscular and vascular integrity. L-citrulline (CIT), recognized as a precursor to nitric oxide (NO), has been demonstrated to enhance endothelial functionality, improve oxygen transport, and increase muscle perfusion, while leucine has been shown to stimulate muscle protein synthesis. Furthermore, exercise serves to modulate both NO availability and anabolic signaling pathways, thereby amplifying the effects of these amino acids. Recent clinical and experimental research indicates that the concurrent administration of CIT and leucine supplementation, in conjunction with structured exercise regimens, yields superior enhancements in muscle mass, vascular reactivity, and physical performance compared to isolated interventions alone. The aforementioned synergistic effects are facilitated through a comprehensive regulation of mitochondrial biogenesis, alongside a reduction in inflammation and oxidative stress. This review consolidates existing empirical evidence regarding the collective contributions of CIT, leucine, and physical exercise in fostering healthy aging, while also delineating prospective research avenues for the formulation of personalized nutritional and physical strategies aimed at enhancing both muscular and vascular well-being in the elderly population.

## 1 Introduction

The phenomenon of aging is characterized by a gradual deterioration in skeletal muscle mass, strength, and functional capacity, a pathological state referred to as sarcopenia, which plays a substantial role in the development of frailty, metabolic impairment, and the erosion of autonomy among elderly populations. This multifaceted process encompasses various factors, including anabolic resistance, mitochondrial dysfunction, oxidative stress, persistent low-grade inflammation, and compromised vascular perfusion of skeletal muscle (1, 2). Historically, sarcopenia has been predominantly conceptualized as a disorder centered on muscle; however, emerging research underscores the pivotal importance of muscle–vasculature interaction in preserving muscle integrity throughout the aging process. The intricate structural and biochemical interactions between skeletal muscle and the vascular endothelium facilitate optimal nutrient and oxygen transport, the clearance of metabolic byproducts, and the release of myokines and vasculokines that mutually regulate their respective functions (3, 4).

As individuals age, the bidirectional communication between muscle and vascular systems becomes compromised, resulting in a reduction of capillary density, a decrease in the bioavailability of endothelial nitric oxide (NO), and an overall decline in muscle perfusion—these elements collectively contribute to the intensification of anabolic resistance and the acceleration of sarcopenic progression (5, 6). Within this framework, integrative approaches that combine nutritional and exercise interventions have garnered significant scholarly attention as pivotal strategies to mitigate age-associated declines in both muscular and vascular functionalities. Physical activity, particularly through resistance and aerobic training modalities, catalyzes angiogenesis, augments endothelial functionality, and stimulates anabolic signaling cascades, thereby enhancing muscle protein synthesis (MPS) and metabolic adaptability (7, 8). The incorporation of targeted nutritional support alongside exercise regimens may further amplify these beneficial effects. Among the various nutritional interventions, L-citrulline (CIT) and leucine have emerged as noteworthy bioactive compounds that serve to connect the domains of muscle and vascular health.

CIT, classified as a non-essential amino acid and a precursor to L-arginine, has been shown to facilitate the production of NO and promote vasodilation, consequently enhancing endothelial functionality and muscle perfusion (9–11). Concurrently, leucine, which is categorized as a branched-chain amino acid, serves as a powerful activator of the mechanistic target of rapamycin complex 1 (mTORC1) signaling pathway, thereby promoting MPS and mitigating the effects of muscle atrophy (12, 13). Considering the interconnected molecular pathways that associate vascular and muscular health, strategically targeting the muscle–vasculature axis through a combined regimen of CIT and leucine supplementation alongside exercise may present a synergistic strategy for the preservation of muscle metabolism, enhancement of vascular function, and prevention of sarcopenia in the context of aging. Although the branched-chain amino acids (BCAAs) including leucine, isoleucine, and valine, all contribute to muscle metabolism, leucine was selected as the primary focus of this review because it exerts markedly stronger anabolic and signaling effects compared with the other two BCAAs. Leucine functions as the principal nutrient sensor for mTORC1 activation through Sestrin2 and leucyl-tRNA synthetase, leading to a robust stimulation of muscle protein synthesis and partial reversal of anabolic resistance in older adults (12). In contrast, isoleucine and valine largely serve as metabolic substrates and display weaker activation of translational signaling. Therefore, leucine represents the BCAA with the most compelling mechanistic and clinical evidence relevant to muscle–vascular interactions and healthy aging, justifying its emphasis in the present review.

This review is intended to investigate the integrative roles of CIT, leucine, and exercise in the modulation of muscle–vascular interactions, elucidating the underlying molecular mechanisms, and emphasizing their potential implications within the framework of healthy aging and personalized preventive approaches.

## 2 Muscle–vasculature crosstalk in aging: an integrative perspective

### 2.1 Physiological interdependence between skeletal muscle and vascular endothelium

The skeletal musculature and vascular systems exhibit a complex interdependence, constituting a dynamic physiological network that guarantees optimal tissue oxygenation, nutrient transport, and metabolic equilibrium. This interplay between muscle and vasculature encompasses bidirectional communication facilitated by mechanical, biochemical, and molecular signaling pathways that synchronize muscle metabolism with vascular functionality (14, 15). The vascular endothelium modulates muscle perfusion through NO–mediated vasodilation, whereas skeletal muscle subsequently secretes myokines such as interleukin-6 (IL-6), irisin, and vascular endothelial growth factor (VEGF), which influence endothelial cell activity, angiogenesis, and vascular remodeling (16, 17). This reciprocal signaling mechanism upholds the structural and functional coherence of both tissues and is pivotal for adaptive responses to physical exercise, nutrient availability, and metabolic challenges.

### 2.2 Impact of aging on endothelial dysfunction, perfusion, and muscle metabolism

As individuals age, this meticulously regulated system becomes disrupted. Endothelial dysfunction, recognized as a critical feature of vascular aging, is marked by a reduction in NO bioavailability, an elevation in oxidative stress, and a diminished responsiveness to vasodilatory stimuli (18, 19). The disruption of endothelial homeostasis results in compromised perfusion of skeletal muscle, a decrease in capillary density, and inadequate delivery of oxygen and nutrients to muscle fibers, ultimately leading to anabolic resistance and muscular atrophy (20). Simultaneously, skeletal muscle in the elderly demonstrates mitochondrial dysfunction, encompassing diminished mitochondrial biogenesis, compromised oxidative phosphorylation, and an augmented production of reactive oxygen species (ROS), which further intensify endothelial oxidative damage and inflammatory responses (21, 22). This establishes a detrimental cycle wherein vascular and muscular deficit mutually reinforce one another, thereby hastening the progression of sarcopenia.

### 2.3 Role of impaired NO signaling and mitochondrial dysfunction in sarcopenia

At the molecular level, the reduction in NO signaling constitutes a crucial element that correlates vascular dysfunction with the progression of sarcopenia. NO generated by endothelial nitric oxide synthase (eNOS) not only facilitates vasodilation but also exerts a significant influence on muscle metabolism by enhancing mitochondrial efficiency, facilitating glucose uptake, and promoting the activation of satellite cells (23, 24). Age-related declines in eNOS expression and functionality, together with the heightened oxidative degradation of NO by superoxide, undermine these advantageous effects, resulting in metabolic rigidity and muscle atrophy (19). Moreover, compromised mitochondrial signaling reduces energy production and elevates ROS, which subsequently inflict damage upon endothelial cells and inhibit NO synthesis, thereby exacerbating the muscle-vascular dysfunction (25). Together, these findings underscore that sarcopenia transcends a mere muscular ailment and manifests as a systemic vascular–metabolic syndrome, wherein endothelial dysfunction and mitochondrial deterioration serve as converging mechanisms. Consequently, interventions designed to restore NO bioavailability, enhance mitochondrial functionality, and improve microvascular perfusion represent promising therapeutic strategies to mitigate age-related muscular degeneration and sustain physical performance in the elderly population.

## 3 CIT: mechanisms and benefits in aging muscle and vasculature

**Figure 1:**
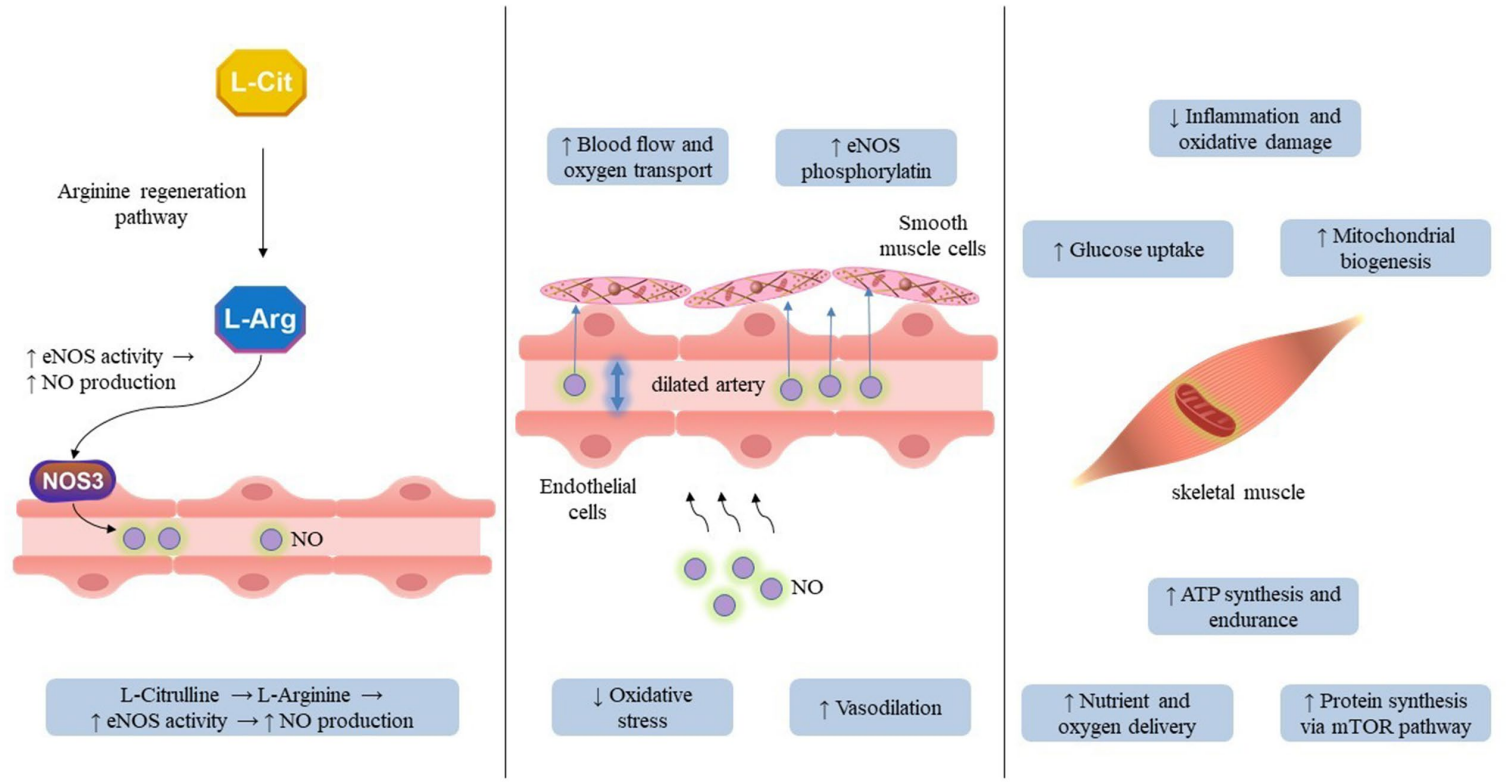
Integrative effects of L-citrulline on muscle–vascular interactions in aging. This figure illustrates the coordinated effects of L-citrulline on nitric-oxide–mediated endothelial function, muscle perfusion, mitochondrial activity, and metabolic regulation. The vertical lines are used solely to organize the visual layout and do not represent conceptual separation between components. The pathways should be interpreted horizontally, highlighting the continuous interaction between vascular improvements and downstream muscular adaptations. Arrows indicate mechanistic links contributing to enhanced perfusion, oxidative capacity, and muscle function in older adults.

Aging is characterized by a gradual deterioration in skeletal muscle mass, strength, and vascular functionality which primarily linked to mitochondrial dysfunction, oxidative stress, endothelial dysfunction, and persistent low-grade inflammation. Within this framework, CIT, a non-essential amino acid and a critical intermediary in the urea cycle, has emerged as a noteworthy nutritional agent with diverse advantages for the preservation of muscular and vascular integrity in the context of aging (26, 27). From a mechanistic perspective, CIT functions as a precursor to L-arginine, which subsequently stimulates the production of NO via the activation of eNOS. In contrast to the direct supplementation of L-arginine, which is subject to significant hepatic metabolism, CIT circumvents first-pass degradation and represents a more effective approach for enhancing systemic L-arginine levels and the bioavailability of NO. An augmented synthesis of NO fosters vasodilation, endothelial restoration, and enhanced blood circulation, thus mitigating the vascular rigidity and compromised perfusion typically associated with aging tissues. In skeletal muscle, augmented NO signaling has been demonstrated to enhance the delivery of nutrients, promote mitochondrial biogenesis, and facilitate glucose uptake, thereby contributing to improved muscle oxygenation and endurance (28). Moreover, CIT plays a crucial role in the modulation of ammonia detoxification and supports protein synthesis through the mTOR signaling pathway, which may serve to alleviate age-associated sarcopenia. Empirical investigations reveal that the supplementation of CIT can promote MPS, particularly when administered in conjunction with resistance training, indicating a potential synergistic effect on anabolic signaling pathways. From a redox perspective, CIT demonstrates antioxidant and anti-inflammatory properties, partly through the reduction of ROS derived from NADPH oxidase and the enhancement of glutathione metabolism. These mechanisms contribute to the preservation of endothelial function and mitochondrial integrity, both of which are essential for sustaining vascular elasticity and muscle contractility in the aging population. Clinically, the administration of CIT has been correlated with enhancements in endothelial function, arterial compliance, and exercise performance among geriatric populations as well as individuals suffering from metabolic or cardiovascular pathologies (29, 30). By augmenting both vascular reactivity and the efficiency of skeletal muscle, CIT provides a dual therapeutic pathway to mitigate the functional deterioration associated with the aging process (Figure 1).

Several studies demonstrate that combining CIT supplementation with high-intensity interval training (HIIT) yields greater functional benefits in older adults than HIIT alone. In dynapenic-obese elderly individuals, CIT enhanced the effects of HIIT by producing superior gains in Timed Up & Go performance and upper-limb strength. Similar results were observed in healthy older adults, where a 12-week HIIT+CIT intervention led to greater reductions in BMI and android fat mass, alongside larger improvements in gait speed, compared with HIIT plus placebo. Collectively, these findings suggest that CIT may potentiate HIIT-induced adaptations in body composition, mobility, and muscle function in aging populations (31, 32).

Evidence indicates that CIT enhances exercise-induced adaptations in older adults. In obese older individuals, combining CIT with a 12-week HIIT program produced greater benefits than HIIT alone, including larger increases in handgrip and quadriceps strength, reductions in fat mass, and favorable shifts in adipose gene expression linked to lipid metabolism. Similarly, in hypertensive postmenopausal women, CIT improved leg endothelial function, and its combination with slow-velocity, low-intensity resistance training further augmented vascular dilation, leg lean mass, and muscle strength. These studies collectively demonstrate that CIT acts synergistically with both HIIT and resistance training to improve vascular health, muscle strength, and body composition in aging populations (33, 34).

In postmenopausal women, who often present with increased arterial wave reflection and reduced muscle function, CIT shows meaningful vascular benefits. In one study involving obese postmenopausal females, CIT supplementation and whole-body vibration training (WBVT) were tested alone or in combination. All groups experienced reductions in brachial and aortic blood pressure and increases in plasma NO metabolites, but only the WBVT groups showed significant decreases in augmentation index (AIx), with the CIT + WBVT group demonstrating the greatest improvement. These findings suggest that CIT can enhance the vascular responsiveness elicited by WBVT and may be particularly effective in mitigating arterial stiffness (35). Another trial demonstrated that 4 weeks of CIT supplementation (10 g/day) attenuated excessive hemodynamic responses during isometric handgrip exercise and post-exercise muscle ischemia in postmenopausal women. Compared with placebo, CIT significantly blunted increases in systolic blood pressure, pulse pressure, and forward and backward pressure waves, indicating a reduction in arterial pulsatile load under stress conditions. Together, these studies indicate that CIT improves endothelial function, supports NO-mediated vascular regulation, and reduces cardiovascular strain in postmenopausal women (36).

Metabolomic evidence further supports a synergistic effect of CIT when combined with HIIT in older adults with obesity. In a 12-week trial involving 86 participants, HIIT induced broad shifts in serum metabolites, but CIT supplementation produced more pronounced changes in ten key metabolites. Notably, CIT increased circulating arginine and reduced specific triglycerides and aspartic acid levels, changes that correlated with improvements in fat mass, leptin, and LDL cholesterol. These findings suggest that CIT amplifies HIIT-related metabolic remodeling and that metabolites such as arginine and selected triglyceride species may serve as biomarkers of enhanced cardiometabolic adaptation (37). Complementary evidence comes from a six-week intervention in physically active older adults, where CIT was combined with a structured activity program. Both the CIT and placebo groups improved in strength and endurance; however, CIT produced a significantly greater increase in walking speed, with trends toward improved hormonal and muscle injury markers. These results indicate that CIT may modestly enhance mobility and functional performance beyond physical activity alone, supporting its potential value in mitigating age-related declines linked to inflammation and oxidative stress (38).

Baseline protein intake appears to influence the extent to which older adults benefit from CIT during HIIT. In a 12-week trial involving 73 obese older individuals, all participants improved their functional capacity and body composition, but those with lower protein intake (<1 g/kg/day) experienced distinctly greater benefits when CIT was added. In this subgroup, CIT led to larger reductions in gynoid fat mass and greater increases in leg fat-free mass and handgrip strength compared with both placebo recipients and individuals with higher habitual protein intake. These findings suggest that CIT may be particularly effective in older adults with suboptimal dietary protein, enhancing HIIT-induced improvements in muscle strength and regional body composition (39). Additional evidence comes from a randomized, double-blind trial evaluating chronic supplementation with nitric oxide precursors (nitrate + CIT) in healthy older adults. After 1 month, individuals receiving the combined supplement exhibited a modest but significant reduction in mean arterial pressure and improved exercise efficiency, characterized by lower heart rate and oxygen consumption during submaximal cycling. They also achieved a 5.2% increase in maximal cycling power output, though knee extension strength remained unchanged. Together, these results indicate that chronic NO precursor supplementation may enhance cardiovascular efficiency, aerobic performance, and resting hemodynamics in older adults (40) (Table 1).

**Table 1:** Summary of clinical studies investigating the combined effects of L-citrulline supplementation and exercise on muscle and vascular function in aging populations.

Participants	Disease/condition	Duration	Exercise program	L-citrulline dosage	Measured outcomes	Main findings	References
56 obese and dynapenic older adults (age 65–68 years)	Dynapenic obesity	12 weeks	High-Intensity Interval Training (elliptical trainer, 3×/week)	10 g/day	Body composition, muscle strength, muscle power, functional and aerobic capacity	HIIT + CIT improved upper limb strength and walking speed more than HIIT alone	(31)
44 sedentary, non-obese older adults (>65 years)	Healthy, inactive	12 weeks	HIIT (30 min/session, elliptical, 3×/week; 30 s > 85% HR, 1:50 min at 65% HR)	10 g/day	Body composition, gait speed, muscle strength, energy expenditure, biomarkers	Greater improvements in BMI, android fat, and gait speed with HIIT + CIT vs. HIIT alone	(32)
81 obese older adults (mean age ≈ 67 years)	Obesity, age-related muscle decline	12 weeks	HIIT (elliptical trainer)	10 g/day	Muscle strength, body composition, mitochondrial and adipose tissue markers	HIIT + CIT led to greater increases in muscle strength and fat mass reduction; enhanced mitochondrial quality control and lipid metabolism	(33)
24 hypertensive postmenopausal women	Hypertension, endothelial dysfunction	8 weeks (4 weeks CIT alone + 4 weeks CIT + SVLIRT)	Slow velocity, low-intensity resistance training	10 g/day	Endothelial function (sfemFMD), leg lean mass, muscle strength	CIT alone improved endothelial function; CIT + SVLIRT further increased leg lean mass and strength	(34)
41 obese postmenopausal women	Obesity, vascular dysfunction	8 weeks	Whole-body vibration training	Not specified	Blood pressure, augmentation index (AIx), NOx levels	Combined WBVT + CIT improved arterial stiffness (AIx@75) and increased nitric oxide bioavailability	(35)
22 postmenopausal women	Endothelial dysfunction, exaggerated BP response	4 weeks	Low-intensity isometric handgrip exercise	10 g/day	Aortic SBP, pulse pressure, forward/backward wave amplitude	CIT attenuated increases in aortic SBP, PP, Pf, and Pb during metaboreflex activation	(36)
86 obese older adults	Obesity, metabolic dysfunction	12 weeks	HIIT (elliptical)	10 g/day	Serum metabolites, body composition, metabolic markers	44 metabolites changed; 10 affected more with CIT + HIIT; arginine, TG(16:1/18:1/16:0), and aspartic acid linked to improved adiposity and cardiometabolic health	(37)
44 adults (60–73 years)	Sarcopenia, muscle weakness	6 weeks	Age-adapted physical activity program	Not specified	Strength, endurance, walking speed, hormones	Walking speed improved significantly with CIT; strength and endurance showed positive trends	(38)
73 obese older adults	Obesity, muscle dysfunction	12 weeks	HIIT (elliptical)	10 g/day	Body composition, muscle strength, protein intake stratification	CIT + HIIT improved strength and body composition; greater benefits in low-protein consumers (<1 g/kg/day)	(39)
24 healthy older adults (60–70 years)	Vascular aging, reduced NO bioavailability	4 weeks	Whole-body (cycling, knee extension)	6 g/day (with 520 mg nitrate)	BP, arterial stiffness, exercise performance, oxygenation	Nitrate + CIT reduced BP, improved cycling performance, and enhanced cardiovascular efficiency	(40)
25 older adults (12 men, 13 women)	Healthy, age-related blood flow decline	14 days	Calf exercise	6 g/day	Femoral blood flow, vascular conductance, plasma arginine	Increased blood flow (+11%) and vascular conductance (+14%) in men; no effect in women	(41)
21 elderly men (65–80 years)	Anabolic resistance	Acute, post-exercise	Unilateral resistance exercise	10 g + 15 g protein	MPS, microvascular perfusion, mTORC1 signaling	Citrulline with low protein did not enhance MPS or microvascular perfusion	(42)
12 older males	Hypertension, arterial stiffness	6 days	Isometric knee extension	6 g/day	BP, pulse wave reflection, arterial stiffness	No significant effects on BP or arterial stiffness at rest or during exercise	(43)
26 adults (11 older, 15 young; 15 women, 11 men)	Healthy	7 days, crossover	Treadmill walking at 40% HR reserve	6 g/day	Oxygen uptake, MRT, oxygen deficit	Improved oxygen uptake kinetics in men, not women or older adults; no effect on oxygen cost	(44)
33 older women (≥65 years)	Physical function decline	6 weeks	Multicomponent exercise, 3×/week	3 g/day	6MWT, sit-to-stand, SPPB, vitamin D, blood biomarkers, QoL	Clinically relevant improvements in functional tests and vitamin D; trends toward better metabolic/hormonal responses	(45)
14 healthy older males	Respiratory function, aging	7 days	Incremental resistive breathing	6 g/day	Pulmonary function, MIP, NO•, muscle oxygenation	Increased exhaled NO•, but no effect on respiratory performance or oxygenation	(46)
23 older women (low BMI 16–21 kg/m^2^)	Sarcopenia risk	20 weeks	Weight-bearing + square stepping, 1×/week	0.8 g CIT + 1.6 g LEU, 2×/day	Body composition, physical activity, plasma amino acids	Increased body mass, BMI, PA, and phenylalanine; suggests sarcopenia/frailty prevention	(47)
30 inactive older men	Sarcopenia, age-related muscle decline	8 weeks	HIIT, 2×/week, 30 s cycling at 85–90% HR reserve	8 g single alternate day	Myostatin, body fat, muscle strength, aerobic power	HIT+CIT decreased myostatin, improved strength, aerobic power, and reduced fat mass	(48)
16 middle-aged/older adults (53–72 years)	Type 2 diabetes, endothelial dysfunction	4 weeks (crossover)	Not specified	6 g/day	Endothelial function (LnRHI), muscle microvascular reactivity, handgrip and calf strength	Improved endothelial function, muscle microvascular reactivity, and calf muscle strength	(49)
16 healthy/prediabetic older adults (68.8 ± 9.8 years)	Prediabetes, endothelial dysfunction	2 weeks, crossover	Handgrip and PRET	Not specified	Microvascular function, muscle oxygenation, exercise lipolysis	No significant changes; trends suggest potential positive direction	(50)

HIIT, high-intensity interval training; CIT, L-citrulline; SVLIRT, slow-velocity low-intensity resistance training; WBVT, whole-body vibration training; Aix, augmentation index; NOx, nitric oxide metabolites; SBP, systolic blood pressure; PP, pulse pressure; Pf/Pb, forward/backward pressure waves; FFM, fat-free mass; TG, triglyceride; HR, heart rate; LEU, leucine; MPS, myofibrillar protein synthesis; MRT, mean response time; BP, blood pressure; FVC, femoral vascular conductance; NO•, nitric oxide; 6MWT, 6-min walk test; SPPB, Short Physical Performance Battery; LnRHI, log-transformed reactive hyperemia index; PRET, perceptually regulated exercise test.

Two studies examined the effects of short-term CIT supplementation, which elevates L-arginine (Arg) and supports nitric oxide (NO) production, on vascular and muscle responses in older adults. In a 14-day randomized, double-blind, crossover trial with 25 participants, CIT (6 g/day) significantly increased plasma Arg in both sexes. Older men experienced reduced diastolic blood pressure and improved exercise-induced femoral blood flow (+11%) and vascular conductance (+14%), whereas women showed no such benefits. No changes occurred with placebo. These findings suggest CIT may modestly enhance muscle perfusion and exercise tolerance specifically in older males (41). A second study tested whether CIT could enhance postprandial muscle blood flow, microvascular perfusion, and muscle protein synthesis (MPS) in elderly men following unilateral resistance exercise. Twenty-one participants consumed either high-dose whey (45 g), low-dose whey plus CIT (15 g + 10 g CIT), or whey plus nonessential amino acids. Although CIT raised plasma Arg, it did not improve blood flow, perfusion, or MPS. The highest MPS occurred with high-dose whey alone, indicating that adding CIT to a low-protein dose does not enhance anabolic responses in older adults (42) (Table 2).

**Table 2:** Summary of clinical studies investigating the combined effects of leucine supplementation and exercise on muscle and vascular function in aging populations.

Participants	Disease/condition	Duration	Exercise program	Leucine dosage	Main findings	References
19 pre/frail older women (77.5 ± 1.3 y, BMI 25.1 ± 0.9 kg/m^2^)	Frailty (pre/frail older women)	12 weeks	Resistance training, 3×/week, with optimized protein diet (1.2 g/kg/day)	7.5 g/day leucine vs. 5.1 g/day alanine (placebo)	↑ Myofibrillar fractional synthesis rate (47%), ↑ type 1 and 2a CSA, ↑ lean mass (2%), ↓ Frailty Criteria (64%); no added effect of leucine	(55)
178 pre-frail older adults (112 control, 44 Nu, 22 Nu+Ex)	Pre-frailty	3 months (+3 months follow-up)	Exercise + nutrition (Nu+Ex) group (unspecified exercise type)	Leucine-enriched protein (dose not specified)	↑ Gait speed, 5 × STS, SPPB, depression, perceived health, FFM, ASM; ↓ IL-6 and TNF-α (not sustained at 6 months)	(56)
81 older adults (≥65 y) with sarcopenia	Sarcopenia	24 weeks + 24 weeks de-training	RT (2×/week, supervised)	11 g protein + 2.3 g leucine per serving (twice/week)	↑ ASMI and HGS post-intervention; sustained gains after de-training in RT + PRO group	(57)
41 post-hospitalized older adults	Sarcopenia and frailty	12 weeks	RT program (unspecified frequency)	Leucine-enriched whey protein (dose not specified)	↑ Physical performance and frailty (both groups); no group differences; ↓ myostatin associated with ↑ ASM	(58)
100 community-dwelling older adults (68.7 ± 5.8 y)	Healthy aging/risk of sarcopenia	16 weeks	Resistance (2×/wk) + functional (1×/wk)	1.5 g protein/kg/day (3 ×/day leucine-enriched whey)	↓ LDL-C, insulin, HOMA-IR; ↓ resistin (P only); no change in eGFR; ↑ protein intake to > 1.5 g/kg/day	(59)
123 older adults with obesity and T2DM	Type 2 diabetes + obesity	13 weeks	Combined lifestyle (diet + exercise)	Whey protein + leucine + vitamin D (dose not specified)	↑ Appendicular and total lean mass; ↑ insulin sensitivity (Matsuda index); ↑ leg muscle mass trend	(60)
100 older adults (69 ± 6 y; 52% women)	Healthy aging	16 weeks	Resistance (2×/wk) + functional (1×/wk)	Leucine-enriched whey protein (1.5 g/kg/day; 3×/day)	↓ Muscle fatigue (rectus femoris and biceps femoris); ↑ HR-QOL (E only); no change in mass or fat	(61)
19 prefrail/frail women (77.5 ± 1.3 y)	Frailty and sarcopenia	3 months	RT 3×/wk. + optimized protein diet (1.2 g/kg/day)	7.5 g/day L-leucine vs. alanine placebo	↑ Lean mass, ↓ fat %; no change in insulin sensitivity or REE vs. placebo	(62)
48 healthy older men (66 ± 1 y)	Healthy aging	Single session (acute study)	Single RT bout (leg press, leg extension, pull-down, chest press)	Milk protein with 0, 15, 30, 45 g protein (1-^13^C leucine tracer)	Dose-dependent ↑ in net protein balance and myofibrillar protein synthesis; ≥30 g protein effective	(63)
41 older men (70 ± 1 y)	Healthy, active older adults	12 weeks	Whole-body resistance training, 3×/week	3 g leucine + 21 g protein after exercise and before sleep	Protein supplementation did not further enhance muscle mass, strength, or protein synthesis vs. placebo	(64)
22 older women (69 ± 1 y; 11/group)	Healthy	6 days	Unilateral resistance exercise	3 g leucine + 10 g milk protein (vs. 25 g whey protein with 3 g leucine)	Lower-protein, leucine-matched supplement produced similar acute and integrated myofibrillar protein synthesis as high-protein whey	(65)
22 older women (65–75 y; 11/group)	Healthy	Not specified (short-term)	Unilateral leg resistance exercise	4.2 g leucine + 15 g milk protein (vs. 1.3 g leucine + 15 g mixed protein)	Higher leucine content enhanced both acute and integrated muscle protein synthesis in resting and exercised legs	(66)
24 older women (65 ± 1 y; 8/group)	Healthy	Acute (hours)	6 × 8 unilateral leg extensions (75% 1RM)	0.6 g (1.5 g LEAA), 2.4 g (6 g LEAA), or 40 g whey protein	Even low-dose LEAA (0.6 g leucine) robustly stimulated MPS; higher doses or whey gave no additional benefit	(67)
42 older adults (55–70 y)	Healthy	8 weeks	Not specified (daily activity maintained)	2 g leucine + L-carnitine 1,500 mg + creatine 3,000 mg + Vit D3	Combination increased lean mass, leg strength, and mTOR expression compared with placebo	(68)
18 men (9 young 24 ± 6 y; 9 older 70 ± 5 y)	Healthy	Acute (4 h post-exercise)	6 × 8 knee extensions at 75% 1RM	4.2 g leucine + 10 g protein + 24 g carbohydrate	Leucine-enriched protein drink enhanced post-exercise muscle protein synthesis in both young and older men	(69)
30 older adults (≥60 y)	Healthy	12 weeks	Resistance training	10 g leucine/day	Leucine + training improved leg strength and functional performance more than training alone	(70)
16 older women (66 ± 2.5 y; 8/group)	Healthy	Acute (0–4 h)	6 × 8 knee extensions at 75% 1RM	1.2 g leucine (40% of 3 g LEAA mix)	Low-dose leucine-enriched EAA stimulated muscle protein synthesis equivalently to 20 g whey protein	(71)
15 older men (*n* = 7–8/group)	Healthy	24 h post-exercise	Resistance training	3.5 g leucine vs. 1.85 g leucine (EAA)	Leucine-enriched EAA prolonged muscle protein synthesis and amino acid transporter expression post-exercise	(72)

wk, week; RE, resistance exercise; MPS, muscle protein synthesis; myoPS, myofibrillar protein synthesis; LEAA, leucine-enriched essential amino acids; WP, whey protein; SFO, Slim-Fast Optima; EX, exercise; ALA: alanine.

Hypertension and arterial stiffness are major cardiovascular risk factors, and CIT, a NO precursor, has been investigated as a nutritional strategy to lower blood pressure. In a double-blind, crossover study of 12 older men, participants consumed either 6 g/day of CIT or placebo for 6 days. Researchers measured central and peripheral blood pressure, arterial stiffness, and pulse wave reflection at rest and during isometric knee extension exercise. CIT produced no significant changes in aortic or brachial pressures, pulse pressure, heart rate, augmentation index, or carotid-femoral pulse wave velocity. Thus, short-term CIT supplementation did not reduce blood pressure or arterial stiffness in this population (43). A second line of research evaluated whether CIT can improve oxygen uptake kinetics, given prior evidence of enhanced muscle oxygenation in young adults during cycling. In a 7-day randomized, double-blind, crossover trial involving 26 participants (younger and older adults; both sexes), investigators assessed oxygen uptake, oxygen cost, mean response time (MRT), and oxygen deficit during treadmill walking. CIT did not affect oxygen kinetics in older adults or in women. In younger adults, CIT modestly lowered oxygen deficit, and in males specifically, it significantly improved MRT and reduced oxygen deficit compared with placebo. These results suggest CIT accelerates the onset of oxygen uptake during exercise in younger men but has no measurable effect on moderate-intensity walking in older adults or in women (44).

A randomized, double-blind, placebo-controlled pilot study evaluated 6 weeks of multicomponent exercise training combined with CIT malate supplementation in older women (≥65 years). Thirty-three participants completed the program while consuming either 3 g/day of CIT malate or placebo. Although most between-group differences were not statistically significant, women receiving CIT malate showed clinically meaningful improvements in physical function, including better sit-to-stand performance (*p* = 0.023), a + 61 m increase in 6-min walk distance, and higher SPPB scores. They also displayed modest increases in vitamin D (+3.82 ng/mL) and favorable tendencies in metabolic, hormonal, and quality-of-life measures—particularly in physical and environmental domains. These findings suggest that CIT malate combined with exercise may help preserve functional health and prevent physical decline in older women, warranting further research (45). A second study assessed whether short-term CIT supplementation (6 g/day for 7 days) affects respiratory muscle performance, fatigue, and oxygenation in older adults. Fourteen healthy older men completed a double-blind crossover protocol evaluating pulmonary function, maximal inspiratory pressure, perceived exertion, sternocleidomastoid muscle oxygenation, and exhaled NO. CIT significantly increased exhaled NO levels by 26% (*p* < 0.001), but did not improve respiratory muscle strength, pulmonary function, perceived exertion, or muscle oxygenation at rest or after resistive breathing. Thus, despite increasing NO availability, short-term CIT provided no measurable ergogenic benefit for respiratory function in this population (46).

A 20-week randomized, double-blind, placebo-controlled trial examined the effects of combined CIT (0.8 g) and leucine (1.6 g) supplementation with weekly exercise in older Japanese women with low BMI (16–21 kg/m^2^). The exercise plus CIT–leucine group (*n* = 10) showed significant gains in body weight, BMI, total body mass, household and overall physical activity, and plasma phenylalanine levels compared with controls. These findings suggest that CIT + leucine taken alongside exercise may improve body composition and activity levels, potentially reducing sarcopenia and frailty risk in older women (47). A second study evaluated an 8-week protocol combining low-frequency high-intensity interval training (HIIT) with CIT supplementation in 30 sedentary older men. Participants were assigned to HIIT alone, CIT alone (8 g every other day), or HIIT + CIT. The HIIT + CIT group experienced significant reductions in myostatin and body fat percentage, along with improvements in muscular strength and aerobic capacity relative to the CIT-only group. HIIT alone also improved strength, aerobic power, and body fat compared to CIT alone. Overall, the results indicate that low-frequency HIIT, especially when paired with CIT, enhances muscle function and aerobic fitness, offering a promising strategy to counteract sarcopenia in older adults (48).

**Figure 2:**
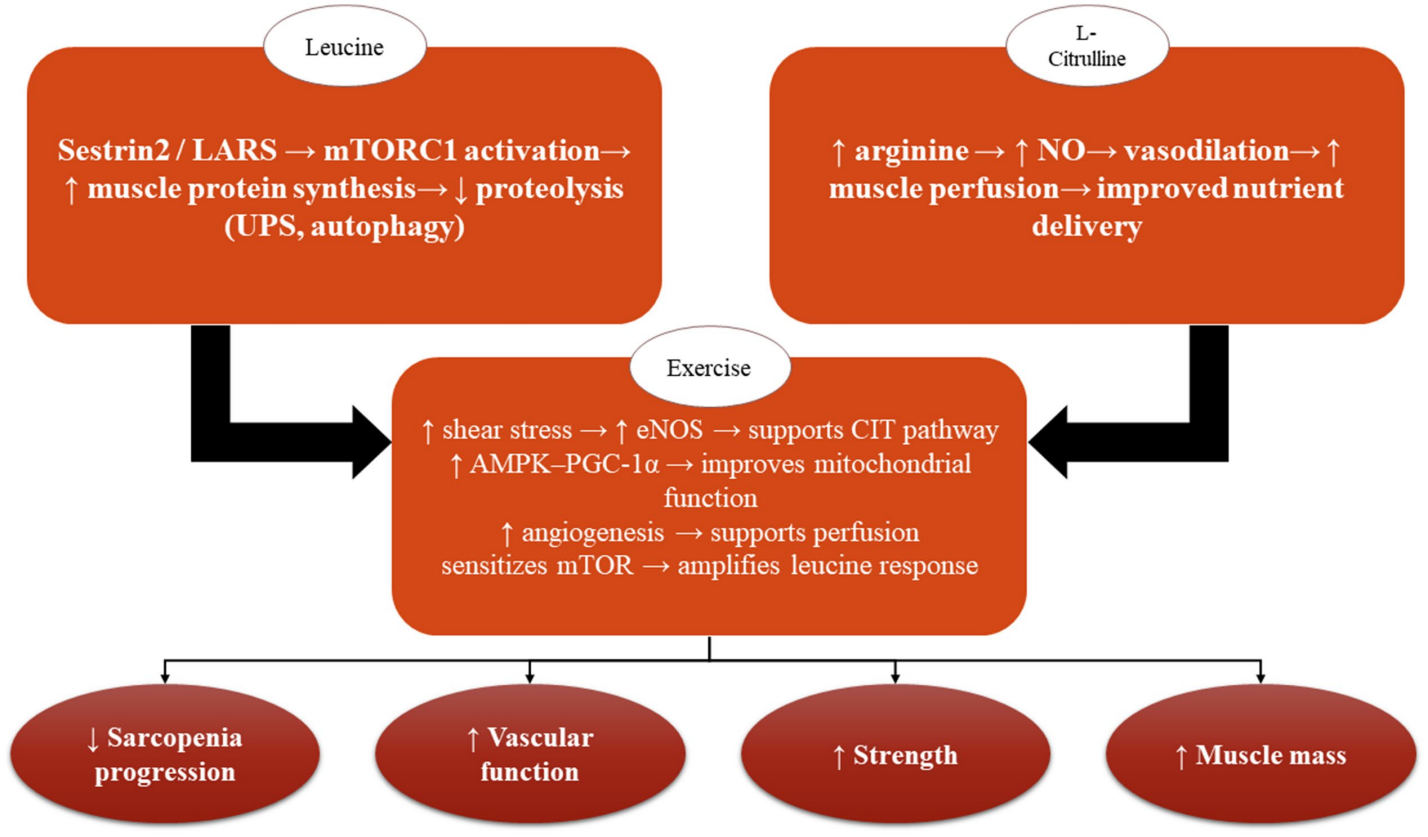
Integrated schematic of L-citrulline, leucine, and exercise pathways in aging muscle and vasculature. This figure provides a synthetic overview of the complementary mechanisms through which L-citrulline, leucine, and physical exercise modulate muscle–vascular function in aging. L-citrulline enhances nitric-oxide (NO) production by increasing arginine availability, leading to improved endothelial function, vasodilation, and skeletal-muscle perfusion. Leucine activates mTORC1 signaling via nutrient-sensing pathways (Sestrin2/LARS), thereby stimulating muscle protein synthesis and attenuating proteolysis. Exercise acts as a central integrator, promoting shear-stress–induced eNOS activation, upregulating AMPK–PGC-1α–mediated mitochondrial biogenesis, and enhancing angiogenesis and metabolic efficiency. Together, these pathways converge to improve nutrient delivery, mitochondrial function, anabolic responsiveness, and ultimately muscle mass and performance in older adults. The figure highlights the interaction between vascular and muscular adaptations and their shared contribution to delaying or attenuating sarcopenia.

A 4-week crossover study examined the effects of CIT supplementation (6 g/day) on microvascular function and muscle strength in 16 middle-aged and older adults with type 2 diabetes. CIT significantly improved endothelial function, forearm microvascular reactivity (TOI indices), plasma arginine levels, and calf muscle strength (both absolute and body-weight–normalized) compared with placebo. Moreover, gains in endothelial function were correlated with increases in calf strength. These findings indicate that CIT supplementation can enhance microvascular health and muscular performance in individuals with type 2 diabetes (49). A separate two-week, double-blind, crossover trial evaluated CIT’s effects on microvascular function, skeletal muscle oxygenation, and exercise-induced lipolysis in 16 older adults who were healthy or prediabetic. CIT did not produce significant changes in any measured parameter during submaximal exercise, though small favorable trends were noted. Overall, short-term CIT supplementation showed minimal physiological impact in this population (50). In conclusion, CIT serves as a metabolically active regulator of NO-dependent signaling, mitochondrial function, and protein metabolism, thereby establishing itself as a significant candidate for nutritional interventions aimed at preserving muscle and vascular integrity during the aging process (Figure 2). Future research endeavors that incorporate omics-based biomarkers and tailored supplementation protocols may elucidate its role further within the frameworks of predictive, preventive, and personalized medicine.

## 4 Leucine: mechanistic pathways in muscle anabolism and anti-sarcopenic action

Leucine is classified as an indispensable branched-chain amino acid that occupies a pivotal position in muscle anabolism and exerts anti-sarcopenic effects. It functions not only as a substrate for protein synthesis but also as a signaling molecule, predominantly through the activation of the mTORC1, which serves as a crucial regulator of MPS. The activation of mTORC1 by leucine occurs through sensing mechanisms including Sestrin2 and leucyl-tRNA synthetase, which facilitate the phosphorylation of downstream effectors such as p70 S6 kinase 1 and 4E-BP1, thereby initiating translation and augmenting myofibrillar protein synthesis. In the geriatric population, leucine has the capacity to surmount anabolic resistance, thereby extending MPS and improving net muscle protein balance. Additionally, it synergistically interacts with resistance exercise to enhance MPS, maintain skeletal muscle mass, increase type II fiber size, elevate strength and functional performance, and mitigate the risk of frailty. Moreover, leucine plays a pivotal role in the modulation of muscle proteostasis by suppressing the pathways associated with protein degradation, including the ubiquitin-proteasome mechanism and autophagy, thus preserving a favorable muscle protein equilibrium. Consequently, the attainment of optimal leucine consumption, particularly following physical exertion, is essential for the prevention of sarcopenia and the promotion of muscular health in aging demographics (51).

It is important to emphasize that leucine’s anabolic and anti-sarcopenic effects are contingent upon a balanced metabolic and amino-acid environment. Evidence shows that isolated leucine supplementation becomes ineffective, and may even have unfavorable consequences, when other essential amino acids are limited or when systemic amino-acid dysregulation is present. This consideration is particularly relevant for older adults, who frequently experience malnutrition, reduced protein intake, and disturbances in circulating amino-acid profiles. Ensuring adequate availability of all essential amino acids is therefore critical for leucine-mediated stimulation of muscle protein synthesis and optimal metabolic outcomes (52–54).

**Figure 3:**
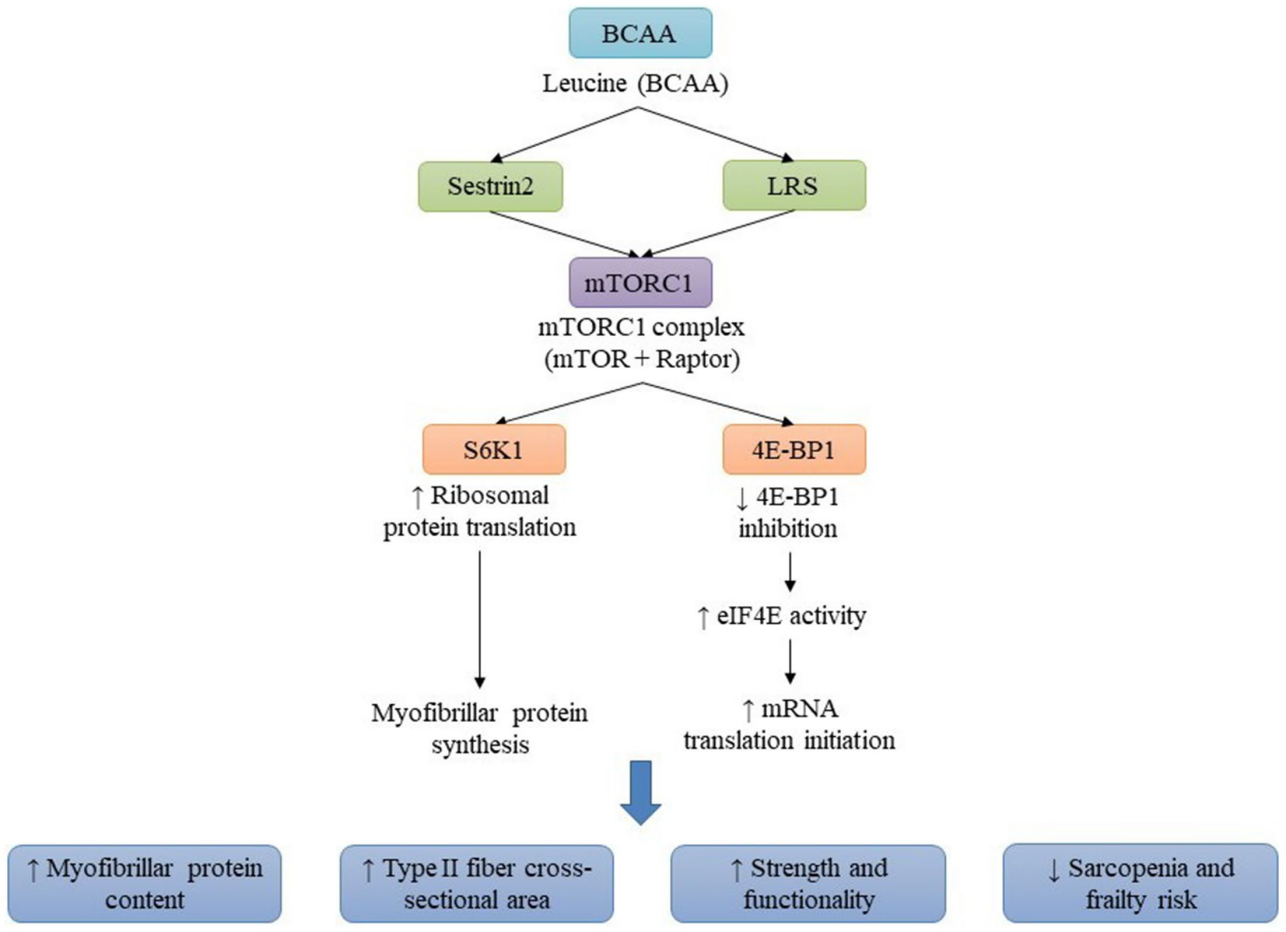
Leucine activates the mTORC1 signaling pathway to promote muscle anabolism and counteract sarcopenia. Leucine binds to Sestrin2 and leucyl-tRNA synthetase, activating the mTORC1 complex, which phosphorylates p70S6 kinase 1 (S6K1) and 4E-BP1. This cascade enhances translation initiation, increases protein synthesis, and suppresses proteolysis and autophagy. The resulting anabolic response improves muscle fiber growth, mitochondrial biogenesis, and overall muscle strength, helping to mitigate age-related anabolic resistance.

Frailty, frequently correlated with sarcopenia, is characterized by diminished muscle strength and mass, which is partially attributable to compromised MPS (Figure 3). A double-blind, placebo-controlled study evaluated 12 weeks of resistance training (RT) combined with optimized protein intake (1.2 g/kg/day) and daily leucine supplementation (7.5 g) versus placebo (alanine, 5.1 g) in 19 pre-frail older women (77.5 ± 1.3 years). RT significantly increased myofibrillar protein synthesis by 47%, enlarged type I and IIa fiber cross-sectional area by 16 and 28%, raised total lean mass by 2%, and reduced frailty criteria by 64%, improving strength and physical function. Although postprandial AKT and S6 phosphorylation rose before training, this response did not persist afterward. Importantly, leucine supplementation provided no additional anabolic benefit, indicating that RT with adequate protein alone is sufficient to enhance muscle mass and functional status in older women (55). A second study by Merchant et al. (56) assessed 3 months of leucine-enriched protein supplementation with or without exercise in 178 pre-frail older adults consuming ≤1 g/kg/day of protein. Participants were assigned to control (*n* = 112), nutrition-only (Nu, *n* = 44), or nutrition plus exercise (Nu+Ex, *n* = 22) groups. At 3 months, the Nu+Ex group showed significant improvements in gait speed, 5 × sit-to-stand performance, SPPB scores, depressive symptoms, perceived health, fat-free mass, and appendicular muscle mass. Both Nu+Ex and Nu groups improved body cell mass and showed decreases in IL-6 and TNF-α. However, these benefits were not maintained at 6 months, highlighting the need for longer-term studies in at-risk populations.

Mori and Tokuda (57) investigated resistance training (RT) with or without leucine-enriched whey protein (PRO) in 90 adults ≥65 years with sarcopenia, followed by 24 weeks of de-training. Participants were assigned to RT + PRO, RT only, or PRO only, receiving biweekly sessions for 24 weeks with controlled energy (≥30 kcal/kg/day) and protein intake (≥1.2 g/kg/day). The RT + PRO group showed significant gains in appendicular skeletal muscle mass index (ASMI) and handgrip strength (HGS), and these improvements remained higher than RT alone after de-training, indicating better long-term retention. The study suggests that combining RT with whey protein may improve sarcopenia outcomes and preserve muscle function after training stops.

Amasene et al. (58), conducted a 12-week study in 41 recently hospitalized older adults, comparing RT with either leucine-enriched protein or placebo. Both groups showed significant improvements in physical performance (*p* < 0.005) and trends toward reduced frailty (*p* < 0.07), with no major differences between groups. Overall myokine levels were unchanged, but reductions in myostatin were linked to increased muscle mass (*p* < 0.05). These findings indicate that RT after hospitalization improves muscle function and reduces frailty, while the role of myokines such as myostatin requires further study.

The Liverpool Hope University-Sarcopenia Aging Trial (LHU-SAT) conducted an investigation into the impact of leucine-enriched whey protein supplementation, both in isolation and in conjunction with resistance-based exercise, on cardiometabolic health among a cohort of 100 older adults (mean age 68.7 ± 5.8 years). Participants were assigned randomly to one of four groups: control (C), exercise (E), exercise plus protein (EP), or protein-only (P) for a duration of 16 weeks. The intake of protein was elevated to 1.55 g/kg/day in the EP group and 1.93 g/kg/day in the P group. Both the EP and P groups exhibited statistically significant reductions in LDL cholesterol, serum insulin, and HOMA-IR when compared to the control group, with a notable decrease in serum resistin observed exclusively in the P group. No detrimental effects on renal function were noted throughout the study. The results demonstrated that leucine-enriched whey protein supplementation, whether administered alone or in combination with resistance exercise, has a beneficial effect on critical cardiometabolic markers in older adults, thereby endorsing its application in interventions aimed at enhancing metabolic health (59). In another work, Memelink et al. (60) evaluated whether a whey protein drink enriched with leucine and vitamin D could preserve muscle mass and improve glycemic control in 123 older adults with obesity and type 2 diabetes participating in a lifestyle program (diet + exercise). Participants received either the fortified protein drink or an isocaloric control beverage. Muscle mass was measured by DXA, and glycemic control via oral glucose tolerance testing. Compared with controls, the experimental group showed a small, nonsignificant increase in leg muscle mass (+0.28 kg), but significant gains in appendicular muscle mass (+0.36 kg) and total lean mass (+0.92 kg). Insulin sensitivity also improved (Matsuda index +0.52). These findings suggest that enriched protein supplementation may support muscle preservation and metabolic health in this population.

A 16-week randomized controlled trial evaluated physical exercise with or without leucine-enriched whey protein in 100 older adults (mean age 69 ± 6). Participants were assigned to control (C), exercise (E), exercise + protein (EP), or protein-only (P). The E and EP groups completed resistance and functional training, while EP and P received 1.5 g/kg/day of protein. After the intervention, both E and EP groups showed improved fatigue resistance in the rectus femoris and biceps femoris, and only the E group showed gains in health-related quality of life (HR-QOL). Muscle and fat mass remained unchanged across groups. The findings indicate that exercise improves muscle fatigue resistance and HR-QOL, while added protein offers no extra benefit when baseline protein intake is already adequate (61). Jacob et al. (62) examined whether adding leucine to resistance training (RT) improves insulin sensitivity in 19 nondiabetic prefrail and frail older women (mean age 77.5 ± 1.3; BMI 25.1 ± 0.9). Participants completed 3 months of RT (3×/week) with a protein-optimized diet (1.2 g/kg/day) and received either 7.5 g/day leucine or an alanine placebo. RT increased lean body mass and reduced body fat in both groups, but leucine had no significant effect on fasting or postprandial glucose or insulin, nor on resting energy expenditure. The study concludes that leucine supplementation does not improve insulin sensitivity when combined with RT and adequate dietary protein.

Holwerda et al. (63) investigated protein dose–response effects on post-exercise protein metabolism in 48 healthy older men (mean age 66 ± 1; BMI 25.4 ± 0.3). After a single RT session, participants consumed 0, 15, 30, or 45 g of milk protein. Using stable isotope tracers, researchers found dose-dependent increases in whole-body net protein balance and incorporation of dietary amino acids into myofibrillar protein. Myofibrillar protein synthesis rose significantly with 30 g and 45 g doses versus 0 g. The study concludes that post-exercise protein is rapidly digested and absorbed, and ≥30 g is required to effectively stimulate myofibrillar protein synthesis in older men (63).

Holwerda et al. (64) determined the efficacy of protein supplementation administered subsequent to exercise and prior to sleep in augmenting muscle mass and strength adaptations during resistance training among a cohort of 41 older males (mean age 70 ± 1 y, BMI 25.3 ± 0.4). The participants engaged in a regimen of whole-body resistance training (3×/week) and were systematically assigned to receive either a protein supplement (21 g, 3 g leucine) or a placebo matched for energy content. The primary outcomes assessed included the one-repetition maximum (1RM) strength, indicators of muscle hypertrophy (as measured by DXA, CT, and biopsy), and rates of MPS (utilizing deuterated water as a tracer). Both experimental groups exhibited statistically significant increases in leg-extension strength, quadriceps cross-sectional area, and the size of type II muscle fibers, with no discernible differences between the two cohorts. Additionally, the rates of MPS were comparable across both groups. The findings of study confirmed that the administration of protein supplementation post-exercise and pre-sleep does not confer additional enhancements in skeletal muscle mass or strength adaptations in physically active older males participating in resistance training. Another investigation aimed to ascertain whether the incorporation of leucine into a modest protein dosage (10 g milk protein + 3 g leucine) elicits MPS responses that are comparable to those produced by a more substantial whey protein dosage (25 g WPI, 3 g leucine) among healthy older women (mean age 69 ± 1 y, *n* = 11/group). Subjects ingested the supplements bi-daily for a duration of 6 days while engaging in unilateral resistance training. Acute MPS exhibited an increase in both fed and fed + exercise conditions for each supplement, with more pronounced enhancements observed in the exercised limb for the leucine condition compared to WPI. Integrated MPS also demonstrated an elevation in the exercised limb for both supplementation groups, whereas responses in the resting limb remained consistent. These findings indicated that lower-protein beverages, matched for leucine content, can effectively activate both acute and integrated MPS, thereby providing a viable approach to sustain muscle anabolic sensitivity in the aging population (65).

A study in healthy older women (65–75 years; *n* = 11/group) examined the effects of leucine-enriched protein on muscle protein synthesis (MPS). Participants followed a controlled diet (1.0 g/kg/day protein) and consumed either a leucine-enriched milk protein supplement (15 g, 4.2 g leucine; LEU) twice daily or an isonitrogenous control drink containing 1.3 g leucine (CON). Using unilateral leg resistance training, researchers measured acute (hourly) and integrated (daily) MPS. The LEU group showed significantly greater acute MPS in both rested (53% vs. 13%) and exercised (87% vs. 30%) legs compared with CON (*p* < 0.001). Integrated MPS also increased in both legs in LEU (rested: 9%, exercised: 17%), whereas CON improved only the exercised leg (7%). These findings demonstrate that twice-daily leucine-enriched protein substantially enhances MyoPS and may help older women maintain muscle mass (66). In a study by Wilkinson et al. (67), compared muscle protein synthesis (MPS) responses to leucine-enriched essential amino acids (LEAA) versus whey protein (WP) in 24 older women (65 ± 1 y). Participants received 1.5 g LEAA (LEAA_1.5), 6 g LEAA (LEAA_6), or 40 g WP. MPS was measured at baseline, in the postprandial state (FED), and after unilateral leg resistance exercise (FED-EX). WP produced the highest plasma insulin and amino acid levels, though LEAA_6 achieved similar peak amino acid concentrations. Postprandially, all groups showed comparable MPS increases during 0–2 h, while LEAA_6 and WP maintained elevated MPS through 0–4 h; FED-EX further enhanced MPS in all groups. Only WP briefly increased p-p70S6K1. The findings indicate that even a small LEAA dose (1.5 g, 0.6 g leucine) can maximally stimulate MPS, highlighting that amino acid composition, especially leucine content, may be more important than total dose for anabolic responses in older women.

A randomized controlled trial evaluated an 8-week nutritional blend containing L-carnitine (1,500 mg), L-leucine (2,000 mg), creatine (3,000 mg), and vitamin D3 (10 μg) in 42 healthy older adults (55–70 years). Participants received either the full combination, L-carnitine alone, or a placebo. Outcomes included lean body mass (DXA), upper and lower body strength, 6-min walk distance, quality of life, and mTOR protein expression. The combination group showed a significant 63.5-point improvement in composite muscle function (*p* = 0.013) versus placebo, along with increases in total lean mass (+1.0 kg), leg lean mass (+0.35 kg), and lower-leg strength (+1.0 kg). mTOR expression also rose, suggesting enhanced protein synthesis. The study concludes that combining L-carnitine, leucine, and creatine significantly improves muscle mass, strength, and performance in older adults (68). A study investigated whether adding leucine to a protein beverage enhances anabolic responses to resistance exercise (RE) in younger (24 ± 6 y) and older (70 ± 5 y) men. Participants completed knee-extension RE (6 × 8 reps at 75% 1RM) and consumed either a leucine-enriched protein drink (4.2 g leucine; LEU) or an isonitrogenous alanine control (ALA). Muscle biopsies taken before and up to 4 h’ post-exercise measured MPS and p70S6K1 phosphorylation. Both age groups showed significantly greater post-exercise MPS (AUC and peak FSR) with LEU than ALA (*p* < 0.05). p70S6K1 phosphorylation increased in all participants, with larger fold-changes in older men receiving LEU. These findings demonstrate that adding leucine to a modest protein dose substantially boosts anabolic responses to resistance exercise in both younger and older adults (69).

A 12-week study examined whether free leucine supplementation (10 g/day) combined with resistance training (RT) improves strength and function more than RT alone in 30 older adults. Participants were assigned to a leucine group (LG; *n* = 15) or control group (CG; *n* = 15) and were assessed at 4 and 12 weeks for isometric leg strength, functional performance, body composition, diet, quality of life, and depressive symptoms. Twenty-four participants completed the 4-week assessment, and eleven completed the 12-week assessment. The LG showed clinically meaningful improvements in isometric leg strength and performed better than CG on chair stand and timed up-and-go tests. Other outcomes showed no significant differences. Overall, leucine supplementation alongside RT moderately enhanced leg strength and specific functional abilities in older adults (70). A study assessed anabolic responses in older women (66 ± 2.5 y) to 20 g whey protein (WP) or a low-dose leucine-enriched essential amino acid supplement (LEAA; 3 g, 40% leucine), with and without resistance exercise (FED vs. FED-EX). Measures included MPS, albumin protein synthesis (APS), plasma amino acids/insulin, leg/muscle blood flow, and anabolic signaling using isotope tracers and phosphoimmunoblotting. WP raised plasma amino acids and insulin more than LEAA, but neither affected leg blood flow; muscle microvascular flow increased only after FED-EX. Both WP and LEAA stimulated MPS similarly (0–2 h post-feeding), with sustained effects (0–4 h) following FED-EX; APS responses were also comparable. The study indicates that older women show modest anabolic responsiveness to nutrition, and WP offers no clear advantage over LEAA (71).

A study examined the effects of post-resistance exercise supplementation with leucine-enriched essential amino acids (EAAs) on muscle anabolism in elderly men. Participants consumed 10 g EAAs containing either standard leucine (1.85 g) or high leucine (3.5 g) 1 h after exercise, with muscle biopsies collected over 24 h. Both groups showed increased p70 S6 kinase 1 phosphorylation at 2 h, but only the high-leucine group had elevated 4E-binding protein 1 phosphorylation. Myofibrillar protein synthesis (MyoPS) rose ~90% at 5 h in both groups, remaining elevated at 24 h only in the high-leucine group. Amino acid transporter mRNA followed similar patterns. The findings indicate that leucine-enriched EAAs prolong anabolic responses and enhance MPS post-exercise, highlighting their potential to optimize exercise interventions in older adults (72).

Overall, leucine assumes a critical function in facilitating muscle anabolism and combating sarcopenia among the elderly population. By activating the mTORC1 signaling pathway, leucine significantly augments MPS, particularly when synergistically administered with resistance training, thereby aiding in the mitigation of age-related anabolic resistance. A comprehensive review of various studies demonstrates that leucine supplementation can enhance lean body mass, improve muscle strength and functionality, and promote the growth of type II muscle fibers, although the ramifications are most notable in individuals exhibiting suboptimal protein consumption. Consistent intake of leucine-enriched protein, particularly following exercise, constitutes an efficacious nutritional approach to preserve muscle mass, improve functional capabilities, and alleviate frailty in aging demographics (Figure 2).

## 5 Exercise as a modulator of muscle–vasculature interactions

### 5.1 Exercise-induced signaling pathways improving endothelial and muscle function

Exercise constitutes a robust, pleiotropic stimulus that orchestrates adaptive responses in both skeletal muscle and the vascular endothelium, thereby facilitating the restoration and preservation of the muscle–vasculature axis, which tends to deteriorate with advancing age. From a mechanistic perspective, the contraction of muscle fibers and the hemodynamic forces elicited by exercise activate a complex network of intracellular signaling pathways which collectively enhance mitochondrial biogenesis and quality control, stimulate angiogenic processes, and augment eNOS activity along with NO production (73, 74). Furthermore, these pathways contribute to the attenuation of chronic low-grade inflammation and oxidative stress, the enhancement of insulin sensitivity and substrate delivery, as well as the priming of satellite cells for repair and hypertrophy, thereby yielding synergistic improvements in both muscle metabolism and vascular functionality (75).

### 5.2 Role of aerobic, resistance, and HIIT modalities in combating sarcopenia

Diverse exercise modalities yield overlapping yet distinct advantages for the aging muscle-vascular unit. The implementation of aerobic endurance training results in an elevation of shear stress and a chronic enhancement of flow-mediated stimuli, which in turn upregulate the expression of eNOS, increase capillary density, and augment mitochondrial oxidative capability—transformations that contribute to improved resting and exercise muscle perfusion as well as metabolic efficiency (18). Resistance training represents the most efficacious singular intervention for the augmentation of muscle mass and strength among older adults; through the mechanisms of mechanotransduction, it robustly activates the mTORC1 signaling pathway, facilitates MPS, and enhances neuromuscular function—outcomes that, when synergistically combined with enhanced local perfusion, mitigate the effects of sarcopenic atrophy (76, 77). HIIT induces swift and substantial enhancements in both endothelial function and mitochondrial capacity, even with a reduced total exercise volume, thereby establishing it as a time-efficient strategy to enhance vascular responsiveness and oxidative metabolism among older populations (78, 79). Pragmatically, mixed programs that combine aerobic and resistance elements maximize gains across perfusion, metabolism, and strength domains important for sarcopenia prevention.

### 5.3 Exercise-mediated regulation of NO bioavailability, angiogenesis, and mitochondrial dynamics

At the convergence of these physiological adaptations, physical exercise facilitates significant modifications in NO bioavailability, angiogenesis, and mitochondrial dynamics. Recurrent elevations in laminar shear stress associated with both aerobic and interval training augment eNOS phosphorylation and subsequent NO release, thereby enhancing vasodilation, capillary recruitment, and nutrient exchange within muscle microvascular networks (18). Furthermore, exercise promotes the upregulation of angiogenic factors mostly prominently VEGF from myocytes and endothelial cells, thereby fostering capillary proliferation and remodeling that are essential for sustained long-term perfusion (17). Simultaneously, physical activity initiates mitochondrial biogenesis (mediated by peroxisome proliferator-activated receptor gamma coactivator 1-alpha, PGC-1α), enhances mitophagy, maintains the balance between mitochondrial fusion and fission, and optimizes electron transport chain efficiency, which collectively mitigates ROS production and safeguards the functionality of both muscle and endothelial cells (74). These synergistic outcomes disrupt the maladaptive cycle characterized by inadequate perfusion → mitochondrial dysfunction → oxidative stress, which is a fundamental contributor to the progression of sarcopenia.

### 5.4 Synergistic effects of exercise with nutritional interventions

Exercise exhibits a potent synergy with nutritional interventions, significantly enhancing muscle-vascular recovery. The intake of protein contemporaneously with resistance training enhances mTOR-mediated translation and overall protein synthesis, thereby promoting hypertrophy and strength development that are considerably more challenging to achieve through either nutrition or exercise in isolation (80). Similarly, dietary approaches that increase NO precursors or mitigate oxidative stress (such as dietary nitrate, CIT/L-arginine, and antioxidant polyphenols) can improve exercise-induced enhancements in endothelial function and microvascular blood flow, thereby augmenting substrate delivery to trained musculature and enhancing training responsiveness in geriatric populations (81). Collectively, exercise serves as a principal regulator of muscle-vascular health; when integrated with specific nutritional strategies, it presents a pragmatically appealing, synergistic approach to preventing or mitigating sarcopenia in aging demographics.

## 6 Integrative roles of CIT, leucine, and exercise: molecular and functional synergy

### 6.1 Shared and distinct molecular targets (NO–mTOR–AMPK axis)

The synergistic interplay of CIT, leucine, and physical exercise constitutes a formidable integrative methodology aimed at reinstating muscle-vascular homeostasis during the aging process by strategically engaging overlapping and complementary molecular pathways. These interventions predominantly intersect at the NO–mTOR–AMPK signaling axis, which represents a pivotal regulatory network that harmonizes muscle metabolism, vascular functionality, and cellular energy equilibrium. CIT functions as a precursor to L-arginine, thereby facilitating the synthesis of NO through the activation of eNOS, which in turn enhances endothelial functionality, vasodilation, and the perfusion of skeletal muscle (11). Concurrently, leucine is recognized as a potent stimulator of the mTORC1 pathway, thereby fostering translational initiation, MPS, and the inhibition of proteolysis (13). Physical exercise concurrently stimulates AMPK and PGC-1α, thereby augmenting mitochondrial biogenesis, oxidative metabolism, and angiogenesis, while also rendering muscle tissue more responsive to nutrient-mediated anabolic signaling (74). Together, these factors form an integrated molecular network in which NO-mediated perfusion facilitates nutrient delivery, leucine-driven mTOR activation supports muscle anabolism, and exercise-induced AMPK signaling maintains metabolic efficiency—collectively counteracting the anabolic resistance associated with aging.

### 6.2 Enhancement of muscle perfusion, nutrient delivery, and protein synthesis

The functional outcomes of this synergistic interaction are manifested in enhanced muscle perfusion, improved nutrient assimilation, and elevated protein synthesis. The increased bioavailability of NO through CIT supplementation serves to optimize microvascular recruitment and the transport of glucose and amino acids, thereby fostering a conducive environment for leucine-mediated activation of the mTOR and exercise-induced metabolic adaptations (49, 82). Physical exercise contributes to an increase in capillary density and mitochondrial turnover, thus facilitating energy-intensive processes such as the accretion of myofibrillar protein and the maintenance of contractile function (83, 84). By integrating vascular and muscular adaptations, this triadic relationship enhances substrate delivery efficiency, expedites recovery, and sustains muscle quality in the aging population. The metabolic interplay between the vascular and skeletal systems thus emerges as a therapeutic focal point, in which CIT enhances endothelial responsiveness, leucine promotes muscle anabolic processes, and exercise serves to integrate both systems through hemodynamic and molecular stimuli.

### 6.3 Impact on myokine and adipokine signaling in the muscle–vascular network

Beyond mere direct metabolic implications, this integrative framework modulates the signaling pathways of myokines and adipokines, thereby reinforcing the endocrine aspect of the interactions between muscle and vascular systems. Engagement in physical exercise incites the secretion of myokines such as irisin, IL-6, and VEGF, which facilitate angiogenesis, enhance insulin sensitivity, and regulate inflammatory processes (17). Furthermore, a decline in pro-inflammatory adipokines such as TNF-α and resistin, alongside an elevation in adiponectin levels, contributes to enhanced metabolic flexibility and improved vascular tone (85). Taken together, these interactions elucidate a systemic endocrine communication that extends the advantages of CIT, leucine, and physical exercise beyond localized muscle metabolism to encompass overarching cardiometabolic health.

### 6.4 Emerging evidence on combinatory interventions in elderly populations

Emerging clinical evidence substantiates the translational potential of integrating these interventions within geriatric cohorts. Clinical trials have elucidated that CIT supplementation enhances endothelial functionality, muscle oxygenation, and exercise performance among older adults (86, 87). The intake of leucine or leucine-enriched protein, particularly when strategically aligned with resistance training, markedly augments MPS and functional strength in comparison to exercise in isolation (80). Moreover, multimodal interventions that amalgamate aerobic and resistance training alongside amino acid supplementation have been demonstrated to enhance muscle mass, ambulation speed, and vascular health in frail elderly populations (88, 89). Such findings accentuate the molecular and functional synergy inherent in targeting NO–mTOR–AMPK pathways through a comprehensive CIT–leucine–exercise approach, thereby providing a robust, individualized framework for the prevention of sarcopenia and the promotion of healthy aging.

Recent clinical evidence further supports the synergistic interaction between CIT supplementation and exercise on vascular and muscle adaptations in aging populations (34). In hypertensive postmenopausal women, a group characterized by high prevalence of endothelial dysfunction and accelerated declines in muscle mass, 4 weeks of CIT supplementation significantly improved superficial femoral artery flow-mediated dilation, and these vascular benefits were amplified when CIT was combined with slow-velocity, low-intensity resistance training. Notably, the combined intervention (CIT + SVLIRT) also resulted in meaningful increases in leg lean mass and muscle strength compared with exercise alone. These findings reinforce the concept that CIT enhances exercise-induced improvements in both perfusion and anabolic responsiveness, underscoring its potential role as a complementary strategy to optimize muscle–vascular health in older adults (34).

In another recent trial, 81 obese older adults completed a 12-week HIIT program on an elliptical trainer, combined with either CIT supplementation or placebo (33). Functional performance, muscle strength, muscle power, body composition, and waist circumference were evaluated before and after the intervention, and a subset underwent muscle and adipose tissue biopsies to assess mitochondrial and lipid-metabolism markers. Both HIIT groups demonstrated significant improvements in functional capacity, total and leg lean mass, muscle power, and reductions in waist circumference. However, only participants receiving CIT exhibited significant reductions in fat mass and greater gains in handgrip and quadriceps strength. At the molecular level, both groups showed increases in markers of mitochondrial content, fusion, and mitophagy, whereas a reduction in the lipid-droplet–associated gene CIDEA was observed exclusively in the HIIT-CIT Group. Overall, these findings indicate that while HIIT alone effectively enhances body composition and physical function in obese older adults, the addition of CIT provides further benefits by promoting greater muscle strength and reducing fat mass, supporting HIIT-CIT as a promising combined strategy to improve metabolic and functional health in this population (33).

Additional evidence underscores the importance of leucine availability for optimizing the anabolic response to both feeding and exercise in older adults (66). In a controlled trial involving healthy older women, twice-daily consumption of a protein beverage enriched with ~4 g of leucine elicited significantly greater increases in both acute and integrated myofibrillar protein synthesis compared with an isonitrogenous mixed-protein beverage containing lower leucine content. Notably, the leucine-rich supplement enhanced protein synthesis in both rested and exercised muscle, whereas the lower-leucine control increased integrated synthesis only in the exercised limb. These findings highlight leucine’s capacity to potentiate basal and exercise-stimulated anabolic signaling, supporting the concept that higher-leucine protein formulations may help attenuate age-related declines in muscle mass when consumed habitually (66).

## 7 Targeting molecular pathways: from mechanisms to clinical implications

### 7.1 Modulation of oxidative stress, inflammation, and mitochondrial quality control

Targeting the molecular pathways that regulate the muscle-vasculature axis presents a highly promising approach to attenuate the advancement of sarcopenia and associated frailty syndromes in the aging population. Integral to this strategy is the modulation of oxidative stress, inflammation, and mitochondrial quality control, which are intricately linked in the maintenance of muscle and vascular integrity. The aging process is marked by a persistent dysregulation between pro-oxidant and antioxidant mechanisms, resulting in the excessive production of ROS that hinder eNOS functionality, diminish NO bioavailability, and exacerbate endothelial dysfunction (19). Within skeletal muscle, oxidative stress inflicts damage upon mitochondrial DNA, proteins, and lipids, thereby undermining mitochondrial respiration and adenosine triphosphate (ATP) synthesis (83). Simultaneously, the process of aging precipitates a phenomenon known as inflammaging, characterized by a chronic low-grade inflammatory condition mediated by heightened concentrations of IL-6, TNF-α, and CRP, which disrupt anabolic signaling pathways, including mTOR, and inhibit MPS (12, 90). Collectively, these molecular modifications engender a self-reinforcing cycle of mitochondrial dysfunction, oxidative damage, and inflammation that expedites the deterioration of both muscular and vascular systems. Interventions aimed at enhancing mitochondrial quality control (MQC)—which encompasses processes such as biogenesis, mitophagy, and the dynamics of fusion and fission—are essential for reinstating metabolic resilience. Physical exercise and particular nutrients, including CIT and leucine, have demonstrated efficacy in upregulating the signaling pathways of PGC-1α, SIRT1, and AMPK, thereby facilitating mitochondrial turnover and alleviating oxidative stress (74). Enhanced MQC not only promotes sustained ATP production but also mitigates ROS generation, thereby safeguarding both endothelial functionality and muscle contractility. Moreover, improving NO signaling through nutritional precursors like CIT may further augment mitochondrial efficiency and vascular perfusion, highlighting the bidirectional therapeutic potential of targeting shared molecular pathways between muscle and endothelium (11).

### 7.2 Potential for preventing frailty and functional decline in older adults

From a translational perspective, the modulation of these molecular networks possesses substantial clinical ramifications for the prevention of frailty and the mitigation of functional decline in the elderly population. Holistic lifestyle interventions that synergistically incorporate exercise regimens with specific nutritional enhancements can restore anabolic sensitivity, augment muscle perfusion, and decrease systemic inflammation—collectively facilitating improvements in physical function, metabolic health, and overall quality of life (91). For instance, resistance training or combined exercise protocols augmented with leucine-enriched proteins have evidenced enhancements in muscle mass and strength, while NO-enhancing nutrients such as CIT or dietary nitrates have demonstrated favorable outcomes in endothelial function and exercise performance (92). These observations underscore the notion that personalized and preventive nutritional strategies can effectively complement exercise-oriented therapies to address the molecular underpinnings of sarcopenia rather than merely alleviating its superficial manifestations.

### 7.3 Metabolic context in aging: gut microbiota, lipid mediators, and their interaction with exercise and amino acid supplementation

Aging is accompanied by profound metabolic remodeling that influences muscle–vascular health and modulates the responsiveness to nutrients such as CIT and leucine, as well as to physical exercise. Two components of this metabolic environment, gut microbiota composition and lipid mediator (oxylipin) profiles, play increasingly recognized roles in anabolic signaling, endothelial function, inflammation, and muscle adaptation (93). Older adults exhibit notable shifts in gut microbiome diversity and composition, marked by reductions in beneficial taxa and an expansion of pro-inflammatory species. These alterations impair nutrient absorption, reduce the bioavailability of amino acids including leucine, and contribute to chronic low-grade inflammation that exacerbates anabolic resistance and endothelial dysfunction. Dysbiosis is additionally linked to impaired SCFA (short-chain fatty acid) production, compromising intestinal barrier integrity and amplifying systemic inflammatory signaling implicated in sarcopenia progression (94).

Emerging evidence indicates that exercise can beneficially remodel the gut microbiota, enhancing SCFA-producing bacteria, restoring microbial diversity, and improving metabolic flexibility independently of diet (95). These changes may potentiate the effects of CIT and leucine by improving amino acid uptake, reducing inflammaging, and enhancing NO-dependent vascular responses.

Oxylipins, bioactive lipid mediators derived from polyunsaturated fatty acids, play central roles in vasodilation, vasoconstriction, inflammation, immune regulation, mitochondrial function, and muscle repair. Aging is associated with a shift toward more pro-inflammatory oxylipin species, which may impair microvascular perfusion and attenuate anabolic responsiveness (96). These changes contribute to endothelial stiffening and diminished vascular reactivity, partially counteracting the benefits of NO precursors such as CIT. Physical exercise has been shown to independently modulate oxylipin profiles, promoting a shift toward anti-inflammatory and pro-resolving metabolites that support mitochondrial biogenesis, angiogenesis, and muscle protein synthesis. This remodeling is associated with improved endothelial function and enhanced muscle recovery capacity across older populations (97, 98). In parallel, increased production of vasodilatory oxylipins may complement CIT-mediated improvements in NO bioavailability, collectively enhancing perfusion during and after exercise.

Gut-derived metabolites (e.g., SCFAs, indoles, secondary bile acids) and oxylipin signatures converge on metabolic pathways central to the CIT–leucine–exercise synergy. For instance:

SCFAs activate AMPK and improve mitochondrial efficiency, potentially enhancing the response to exercise. Anti-inflammatory oxylipins reduce endothelial oxidative stress, supporting NO production and vasodilation. Improved gut permeability decreases systemic inflammation, facilitating mTOR activation by leucine. These interactions highlight the importance of considering broader metabolic networks when interpreting the clinical and mechanistic effects of CIT, leucine, and exercise. Together, gut microbiota composition and oxylipin biology provide an integrative framework that complements the NO–mTOR–AMPK axis and helps explain inter-individual variability in therapeutic responsiveness (99). Collectively, the metabolic context of aging, characterized by microbiome dysbiosis, altered oxylipin patterns, and systemic inflammation, exerts substantial influence on muscle–vascular crosstalk. Exercise beneficially modulates both microbial and lipid mediator networks, and these improvements may enhance or synergize with the effects of CIT and leucine. Incorporating these metabolic dimensions provides a more complete understanding of the mechanisms supporting healthy aging and sarcopenia prevention.

## 8 Future directions and research gaps

Despite the increasing body of evidence that elucidates the synergistic interactions of CIT, leucine, and physical exercise on the enhancement of muscle and vascular health in the aging population, several methodological limitations persist within the extant literature. The majority of clinical investigations conducted thus far are characterized by limited sample sizes, abbreviated intervention periods, and diverse study designs, thereby complicating the establishment of optimal dosing regimens, timing of administration, and the effective combinations of these therapeutic modalities. Furthermore, disparities in participants’ nutritional status, levels of physical activity, and overall metabolic health frequently obfuscate the findings, highlighting the imperative for more rigorously controlled and standardized research initiatives. Future studies ought to employ multi-omics methodologies, including metabolomics, proteomics, transcriptomics, and microbiome analysis, to elucidate the intricate molecular networks that govern the interactions between muscle and vascular systems. Such comprehensive analyses may facilitate the identification of novel biomarkers that predict individual responsiveness to interventions involving CIT, leucine, and exercise. Additionally, longitudinal studies are warranted to evaluate the durability of these benefits and their potential translation into sustained enhancements in functional independence and quality of life among the elderly demographic. Personalized methodologies signify another pivotal domain of research inquiry. The inherent variability in amino acid metabolism, NO bioavailability, and adaptability to exercise indicates that a universal approach may prove ineffective. Tailored nutrition and exercise regimens have the potential to optimize therapeutic outcomes. Moreover, subsequent research should investigate the prospective contributions of these interventions beyond sarcopenia, particularly in the realms of preventing or alleviating age-related vascular aging, metabolic syndrome, and frailty. By integrating mechanistic insights with individualized clinical applications, forthcoming studies can facilitate the development of comprehensive strategies aimed at fostering healthy aging and extending the healthspan.

## 9 Conclusion

The interaction between skeletal muscle and the vascular system constitutes a crucial factor influencing metabolic health and functional capacity throughout the aging process. An increasing body of mechanistic research suggests that CIT and leucine, via their respective roles in the modulation of NO bioavailability and the activation of the mTOR signaling pathway, yield synergistic effects that bolster muscle anabolism, enhance vascular functionality, and promote oxidative equilibrium. Furthermore, physical exercise intensifies these advantageous outcomes by fostering angiogenesis, facilitating mitochondrial biogenesis, and promoting anti-inflammatory signaling, thus reinforcing the critical crosstalk between muscle and vasculature necessary for optimal aging. From a clinical perspective, the integrated application of these nutritional and exercise regimens demonstrates substantial promise in mitigating sarcopenia, augmenting physical performance, and postponing functional deterioration in the elderly population. Translationally, these holistic interventions resonate with the foundational concepts of preventive and personalized medicine. In summary, directing attention to muscle–vasculature interactions through synergistic nutritional supplementation and systematic exercise emerges as a compelling strategy to enhance muscle health, maintain vascular integrity, and prolong healthspan within aging demographics.
